# Effects of the Malnutrition—Eat Additional Meal (MEAM) Diet on the Serum Levels of Albumin and C-Reactive Protein in Hemodialysis Patients

**DOI:** 10.3390/nu14245352

**Published:** 2022-12-16

**Authors:** Lucyna Kozlowska, Jolanta Gromadzinska, Rafal Zwiech, Zbigniew Zbrog, Wojciech Wasowicz

**Affiliations:** 1 Laboratory of Human Metabolism Research, Department of Dietetics, Warsaw University of Life Sciences, 02776 Warsaw, Poland; 2 Department of Environmental and Biological Monitoring, Nofer Institute of Occupational Medicine, 91348 Lodz, Poland; 3 Dialysis Department, Norbert Barlicki Memorial Teaching Hospital No.1, 90153 Lodz, Poland; 4 B.Braun Avitum Dialysis Center, 93515 Lodz, Poland

**Keywords:** dietary intervention, nutritional status, inflammation

## Abstract

The main objective of this project was to evaluate the efficiency of two kinds of nutritional intervention implemented in hemodialysis patients for 24 weeks (traditional nutritional intervention without a meal served before dialysis for group HG1, and nutritional intervention involving a meal served before dialysis for group HG2), and their impact on nutritional status and serum concentrations of C-reactive protein (CRP). Nutritional status and serum biochemical parameters were analyzed in the control group (CG, *n* = 70) and in two homogeneous groups of patients, HG1 (*n* = 35) and HG2 (*n* = 35). There was an interesting trend in both groups of patients connected with increased intake, mainly of energy and protein. In HG1, the greatest increase in energy intake was observed on Sundays, and in HG2 on the days with dialysis. In HG2, after 24 weeks of the nutritional intervention, an increase in serum albumin (*p* = 0.0157) and a decrease in CRP concentration (*p* = 0.0306) were observed, whereas in HG1 there was a decrease in serum albumin concentration (*p* = 0.0043) with no significant change in CRP concentration. The nutritional intervention applied, called the Malnutrition—Eat Additional Meal (MEAM) diet with an easily digestible meal served before dialysis, was aimed at improving the patients’ nutritional status and the obtained results indicate the need not only for substantial reeducation of hemodialysis patients in the area of their diet, but also for undertaking further research and discussions on the possibility of ensuring adequate meals for hemodialysis patients before the dialysis procedure.

## 1. Introduction

Despite many years of efforts and improvements in medical care and dialysis techniques, the mortality rate among patients treated with hemodialysis is very high, at approximately 20% per year [1]. Therefore, discovering factors that lead to poor dialysis outcomes and their successful long-term treatment is of paramount importance. The main causes of death in dialyzed patients are cardiovascular diseases and ongoing inflammatory processes [2,3,4]. A meta-analysis showed a significant inverse relationship between well-known parameters of nutritional status such as serum albumin concentration and all-cause mortality as well as cardiovascular mortality, whereas serum C-reactive protein (CRP) concentration showed a direct statistically significant correlation with all-cause mortality in hemodialysis patients. This indicates the potential impact of nutritional status and infections on mortality, and emphasizes the huge role of the treatment of malnutrition and inflammation in this group of patients [5]. 

Persistent inflammation, usually measured by the serum concentration of CRP, is connected with multiple factors such as toxic uremic milieu and the dialysis technique [6,7,8,9]. Inflammation contributes to decreased serum albumin levels [10] and is connected with malnutrition, inflammation and atherosclerosis (MIA) syndrome [8,11]. Inflammation has a catalytic effect on hypoalbuminemia through inhibition of its synthesis and induction of catabolism [12], being mainly responsible for the relationship between mortality and hypoalbuminemia [6,13].

The prevalence of malnutrition in hemodialysis patients ranges from 23–73% depending on the studied population and on the analyzed nutritional markers [14,15,16]. Overall, it is estimated that malnutrition remains undiagnosed and untreated in approximately three-quarters of hemodialysis patients [17,18,19].

It was shown that a poorer nutritional status in patients treated with hemodialysis is also a strong predictor of short-term and long-term outcomes, as well as increases morbidity, is related to a higher number of cardiovascular incidences, increases the number of hospitalizations and is connected with a low quality of life [20] while a better nutritional status increases the rate of survival [21].

The basis for the development of malnutrition in hemodialysis patients is multifactorial and is connected with nutritional and non-nutrition mechanisms [22,23,24,25,26].

The relationship between nutritional status and mortality indicates the need to look for ways to effectively combat malnutrition in hemodialysis patients. One of the solutions used is intradialytic nutrition. Protein metabolism studies clearly indicate that an additional simple meal (yogurt, cream and protein-enriched milk powder), oral nutritional supplementation or intradialytic parenteral nutrition (IDPN) administered during the dialysis procedure abolished the negative effect of dialysis on the whole-body protein metabolism [27,28]. There are a number of studies proving that long-term intradialytic oral nutritional supplementation or IDPN can improve the outcome in malnourished patients [29,30,31]. 

Studies with various intradialytic supplementation programs have shown them to improve the subjective global assessment after supplementation containing 355 kcal and prepared from simple ingredients [32], body mass after supplementation with two cans of Nepro^®^ containing 960 kcal [33] and biochemical indicators such as serum albumin and prealbumin after supplementation with a can of Nepro^®^ containing 475 kcal [34]. Tomayko et al. [31], after 6 months of oral supplementation with a whey or soy protein administered before the dialysis session, observed a significant decrease in the pre-dialysis interleukin-6 levels. Unfortunately, as none of the studies examined the eating habits and daily nutrient intake, it is difficult to determine whether the benefits have been brought by the additional protein, other nutrients and/or energy intake. 

Although IDPN reverses the net negative whole-body and skeletal muscle protein balance during hemodialysis, it is rather costly, which limits its use to a great extent. Therefore, serving a traditional meal is a more promising, physiological and affordable intervention in hemodialysis patients. Bearing in the mind the problems connected with meal consumption during dialysis, such as a risk of choking or aspiration, along with infection control and hygiene issues, including transmission of such diseases as hepatitis A, two kinds of nutritional intervention were applied, i.e., implementation of individual nutritional recommendations with a meal given just before the dialysis procedure and traditional nutritional intervention without a meal served before the dialysis procedure. The scientific goal of the project was to evaluate the efficiency of these two kinds of nutritional intervention with and without a meal served before dialysis in terms of reduction of inflammation and improvement of nutritional status, using well-known parameters such as the serum concentration of CRP and albumin.

## 2. Materials and Methods

### 2.1. Work Plan, Study Population and Ethical Approval

This study was conducted from November 2019–September 2020 by the Institute of Human Nutrition Sciences, Warsaw University of Life Sciences (WULS-SGGW), and by the Department of Environmental and Biological Monitoring, Nofer Institute of Occupational Medicine (NIOM), within the project titled: “Searching for prognostic metabolomic markers of nutritional intervention effectiveness in hemodialysis patients.” The research was conducted at the Dialysis Department of Norbert Barlicki Memorial Teaching Hospital No. 1 of the Medical University in Łódź and at the B. Braun Avitum Dialysis Center, Łódź, Poland. The managers of the dialysis stations agreed to cooperate in this project. Nutritional status and serum biochemical parameters were analyzed in the control group (CG) and in 2 homogeneous groups of patients treated with hemodialysis, both in short-term and long-term periods:

Group CG—healthy controls matched by sex, age and BMI (*n* = 70; aged 35–70 years),

Group HG1—patients who were to comply with the dietary recommendations without a meal served before dialysis (*n* = 35; aged 35–70 years),

Group HG2—patients who were to comply with the dietary recommendations with a meal served just before dialysis (*n* = 35; aged 35–70 years).

**Figure 1:**
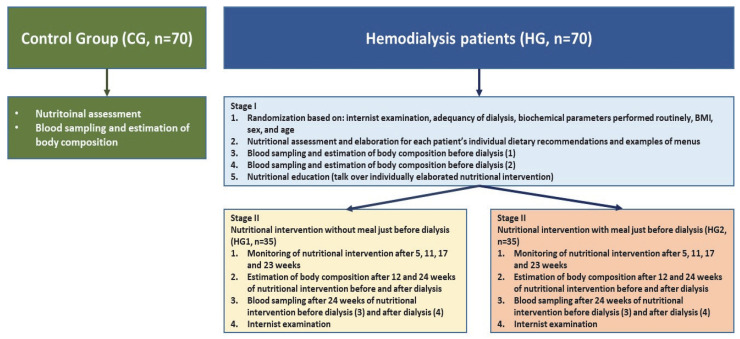
The work plan of the studies: healthy people (control group, CG) and nutritional intervention in 2 homogeneous groups of patients treated with hemodialysis.

The control group consisted of people who were employees of the Institute of Occupational Medicine (this institute was the leader of this project). The selection of patients was conducted in the dialysis stations by internists (a patient without a meal served before dialysis was individually matched to a patient with a meal served before dialysis by age and BMI). A full physical examination was conducted by specialists in nephrology at each time point, with patients evaluated for their symptoms and dialysis-related issues. The work plan of the study is presented in Figure 1.

Briefly, the research plan was as follows: during the first stage of the study (week 0), physical examinations, an assessment of nutritional status as well as an estimation of the body composition and blood sampling were carried out in the groups of hemodialyzed patients, both before and after the dialysis procedures. In the second stage, in both groups of hemodialyzed patients, a 24-week dietary intervention was carried out: in HG1 without a meal just before dialysis and in HG2 with a meal just before dialysis. Patients in HG2 were given meals prepared by a diet catering company. The control group consisted of patients matched with the HG1 and HG2 groups for age and sex. Health status was assessed based on “normal” results of biochemical parameters in blood samples and the body mass index (BMI).

The inclusion criteria for hemodialysis patients were as follows:

- clinically stable patients (men and women), aged 35–70 years, who had undergone hemodialysis for ≥3 months before the study,
- patients undergoing a standard procedure of hemodialysis 3 times/week,
- duration of the hemodialysis session: 180–300 min, depending on the delivered dose of dialysis,
- the surface area of the dialysis membrane: 1.6–2.2 m^2^,
- a dialyzer of the low-flux type.

The exclusion criteria for hemodialysis patients were as follows:

- a BMI of >30 kg/m^2^,
- diabetes type 1 or 2, chronic inflammatory diseases such as rheumatoid arthritis, systemic lupus erythematosus, ankylosing spondylitis, psoriatic arthritis or inflammatory bowel disease, malignancy history in the past 5 years and/or severe cardiac insufficiency (NYHA 4),
- alanine aminotransferase (ALT) and aspartate aminotransferase (AST) ≥ 3× the upper limit of normal (ULN), and total bilirubin ≥1.5 × ULN at randomization.

The study protocol was approved by the Local Ethics Committee in the Nofer Institute of Occupational Medicine in Łódź (protocol No. 08/2018 of 30 May 2018) and it complied with the guidelines of the Declaration of Helsinki. All participants of the study provided their informed consent for participation.

### 2.2. Calculation of Individual Dietary Recommendations for Patients Treated with Hemodialysis

Patients in HG1 and HG2 received dietary recommendations based on nutritional exchangers [35] with individually calculated energy and nutrient values, together with a plan of meals for both days with dialysis and days without dialysis. The European Best Practice Guidelines [36] were incorporated into developing personalized diet plans. The estimation of individual energy demand and nutrient intake was performed as follows: the energy demand was calculated as a product of resting energy expenditure (Harris–Benedict equations) and the activity factor, protein as 1.1 g/kg of an ideal body weight, fat as 33% of energy, carbohydrates as the remaining energy demand, potassium 2730 mg/day, phosphorus 800–1000 mg/day.

### 2.3. Calculation of the Nutritional Value of the Meal Administered just before Dialysis in HG2

Patients in HG2 were given meals prepared by a diet catering company just before dialysis. The nutritional value of meals served just before the dialysis procedure for HG2 was calculated as 20% of the mean energy and nutrient demand in this group of patients. The energy and nutrient value of the served meal was incorporated in the daily energy and nutrient demand—this means that the recommended nutritional values on days with and without the dialysis procedures were the same. This also means that, in both HG1 and HG2, the nutritional value of the daily food rations was calculated in the same way—as described above. 

Meals were delivered to the dialysis stations every day. The products used to prepare them were fresh, of high-quality and with well-defined weight, and each meal was packaged hermetically. The meals were attractive and tasty, e.g., salad with steamed pork loin and colorful lettuce, or chicken with tarragon sauce, pasta and carrot. Foods that are difficult to digest and can lead to the occurrence of gastrointestinal disturbances were excluded from the diet. Fifteen different meal sets were prepared and repeated every 5 weeks. The mean nutritional value of these meals was as follows: energy 416 ± 23 kcal, protein 16.4 ± 0.6 g, fat 14.2 ± 1.6 g, total carbohydrates 58.2 ± 5.6, potassium 452.7 ± 79.6 mg and phosphorus 183.9 ± 10.5 mg.

### 2.4. Nutritional Assessment and Body Composition

Nutrition was assessed by 3-day dietary records: in the control group once (on such days as Friday, Saturday and Sunday) and in hemodialysis patients 5 times—before the nutritional intervention and after 5, 11, 17 and 23 weeks (on days with the dialysis procedure, without the dialysis procedure, and Sunday—neither group had undergone dialysis on that day). This part of the study was performed by qualified personnel. Dieta 6 software (National Institute of Food and Nutrition, Warsaw, Poland) was used to determine daily energy and nutrient intake. Daily energy, protein, phosphorus and potassium intakes were compared to EGPG criteria [36], and intake of other nutrients to the norms for the Polish population [37].

Body composition was measured with the bioimpedance method using the Tanita MC-780MA analyzer (Tanita Corporation, Tokyo, Japan) with 8 stainless steel electrodes and multifrequency current. This method is recommended and widely used in many centers [38]. The measurements in CG were performed once while in both HG1 and HG2 they were performed at 3 time points: before the nutritional intervention and after 12 and 24 weeks (at each time point, measurements were performed before and after the dialysis procedure).

### 2.5. Blood Sampling

Blood samples from patients in HG1 and HG2 (7.5 mL) were collected into S-Monovette^®^ test tubes with a cloth activator, both before the nutritional intervention (week 0) and after 24 weeks (at both time points, blood was drawn before the dialysis procedure). Blood samples from CG were taken once. Serum was separated by centrifugation (10 min, 2000 rpm) and frozen afterwards in Eppendorf tubes at −80 °C until biochemical determinations.

Serum albumin concentrations were determined by the standard laboratory bromocresol green method. Concentrations of CRP were analyzed using the ELISA test performed with the Human C-reactive Protein ELISA Kit (Bioassay Technology Laboratory, Yangpu, China).

### 2.6. Statistical Analysis

All analyses regarding the studied groups, CG, HG1 and HG2, were performed using Statistica software (StatSoft Inc., Tulsa, OK, USA), version 13.1 for Windows. Prior to the analysis, the data were tested for normality using the Shapiro–Wilk test. Statistical significance was established at *p*  <  0.05. Differences between the groups were analyzed using the one-way ANOVA test followed by post hoc Tukey’s test (normal distributed data), and for data without normal distribution with the non-parametric Mann–Whitney U test. Differences between the normal distributed parameters analyzed in subsequent stages of the study were determined using the repeated measures ANOVA test followed by the post hoc least significant difference (LSD) test or using the Wilcoxon signed-rank test for data without normal distribution. 

## 3. Results

The general characteristics of the study participants are presented in Table 1. Overall, the studied groups were composed of 51–60% men, and no significant differences were observed between all the analyzed groups (CG, HG1, HG2) with regard to such parameters as age, height and BMI and no significant differences were observed between all the analyzed groups (CG, HG1, HG2) with regard to such parameters as age (CG vs. HG1 *p* = 0.0521; CG vs. HG2 *p* = 0.01320; HG1 vs. HG2 *p* = 0.6342), height (CG vs. HG1 *p* = 0.3588; CG vs. HG2 *p* = 0.9986; HG1 vs. HG2 *p* = 0.4769) and BMI (CG vs. HG1 *p* = 0.6456; CG vs. HG2 *p* = 0.2217; HG1 vs. HG2 *p* = 0.1678).

With regard to body mass and body composition, no significant differences between HG1 and HG2 were observed in all analyzed weeks in measurements before and after the dialysis procedure. The differences between these parameters in CG and HG1 were not statistically significant, while the only parameter differentiating CG and HG2 was body mass measured after the dialysis procedure, after 12 weeks of the nutritional intervention. Regarding the changes in body weight and body composition during the nutritional intervention in HG1, a significant increase in body weight was observed after 24 weeks of the nutritional intervention compared to baseline values in both pre- and post-dialysis measurements. Additionally, in this group, a significant increase in body weight occurred after 12 weeks of the nutritional intervention in measurements performed after the dialysis procedure. In HG2, there was only a transient increase in total body water after 12 weeks of the nutritional intervention in measurements taken after dialysis, but after 24 weeks there were no significant differences in total body water compared to week 0 (Table 2). 

Table 3 presents data on energy and protein intake (per kg of body mass per day—kcal/kg/day, g/kg/day) in control subjects and hemodialysis patients (HG1 and HG2) during the respective weeks of the nutritional intervention. Given the significant differences in energy and nutrient intake on the days with dialysis (WD), the days without dialysis (WOD) and Sundays (Su), data regarding these days are presented separately. In week 0, the energy and protein intake on the days with dialysis, the days without dialysis and Sundays was significantly lower in both HG1 and HG2 than in CG. In HG1 during the dietary intervention, the energy and protein intake on WOD and on Su (in weeks 17 and 23) did not differ significantly compared to CG, and additionally there were no significant differences in the energy intake on Su in weeks 5 and 11, compared to CG. In contrast, in HG2, energy intake was not significantly different from CG on WD in weeks 17 and 23 of the nutritional intervention. In HG1 there was a significant increase in energy intake on Su in weeks 17 and 23 of the nutritional intervention (*p* = 0.0064, compared to week 0, but also in HG2 on WD in weeks 17 and 23 (*p* = 0.0167). Additionally, in HG1, compared to week 0, there was also a significant increase in protein intake in weeks 5 and 11 (on Su, *p* = 0.0189) as well as in weeks 17 and 23 (on WOD, *p* = 0.0256; on Su, *p* = 0.0159).

**Figure 2:**
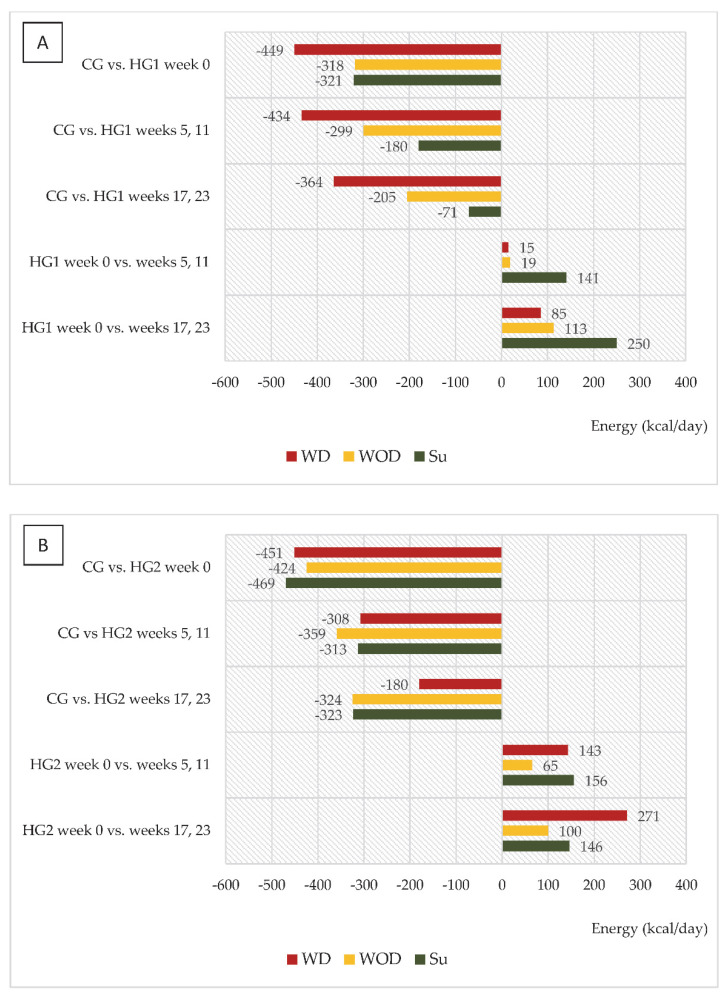
(**A**,**B**) Differences between daily energy intake (kcal/d) in comparison to the control group (CG) and in comparison to baseline values (week 0). (**A**)—data regarding patients who were to comply with the dietary recommendations without a meal served before dialysis (HG1); (**B**)—data regarding patients who were to comply with the dietary recommendations with a meal served before dialysis (HG2); WD—a day with dialysis; WOD—a day without dialysis; Su—Sunday.

**Figure 3:**
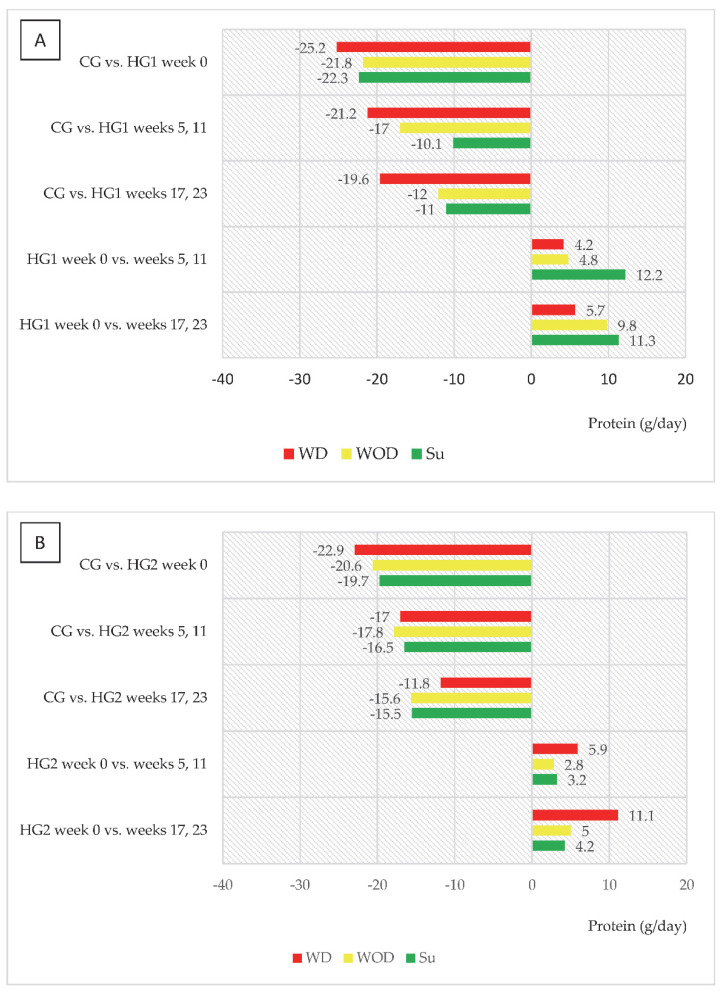
(**A**,**B**) Differences between daily protein intake (g/d) in comparison to the control group (CG) and in comparison to baseline values (week 0). (**A**)—data regarding patients who were to comply with the dietary recommendations without a meal served before dialysis (HG1); (**B**)—data regarding patients who were to comply with the dietary recommendations with a meal served before dialysis (HG2); other abbreviations as in Figure 2.

An interesting trend was observed in both groups of dialysis patients regarding changes in energy and protein intake (kcal/day and g/day, respectively) in relation to intake in CG and to values in week 0 (Figure 2A,B and Figure 3A,B). In HG1, the most marked differences compared to CG were noted on WD, and it was on those days that energy intake increased the least compared to week 0, while the largest increase in intake, reaching 250 kcal/day, was observed on Su (in weeks 17 and 23). For HG2, the largest (characteristic) increase in energy intake was observed on WD, reaching 271 kcal. A similar trend was observed for protein intake in both groups. 

Detailed data on the dietary intake of macronutrients, vitamins and minerals in CG as well as in HG1 and HG2 are presented in the Supplementary Materials (Table S1). It can be generally noted that increased energy intake was also associated with increased consumption of macronutrients, vitamins and minerals. In HG1, in weeks 17 and 24, the consumption of iron, vitamins A, B2, PP, C (on Sundays), B1 (on WOD and on Su) and B6 (on WD and on Su) increased to the extent that it did not differ significantly compared to CG. However, in HG2, in weeks 17 and 24, the intake of fat, vitamin E (on WD) and B1 (on WD, WOD and on Su) did not differ significantly compared to CG. 

The intake of major minerals, such as potassium and phosphorus, both in HG1 and HG2, was within the ranges recommended by EBPG for this group of patients. The calculated average sodium intake exceeded the EBPG recommendations; however, this was due to the fact that in Dieta 6 software (National Institute of Food and Nutrition, Warsaw, Poland) the ready meals contain added salt, but in reality the patients declared that they did not add salt to their food during preparation, which implies that the real sodium intake was lower. As regards nutrition standards for the Polish population [37], in the studied groups of hemodialysis patients, only the dietary intakes of vitamin E and folate were below the recommended dietary allowance (RDA) whereas the intake of other analyzed nutrients was in the range of recommendation.

**Figure 4:**
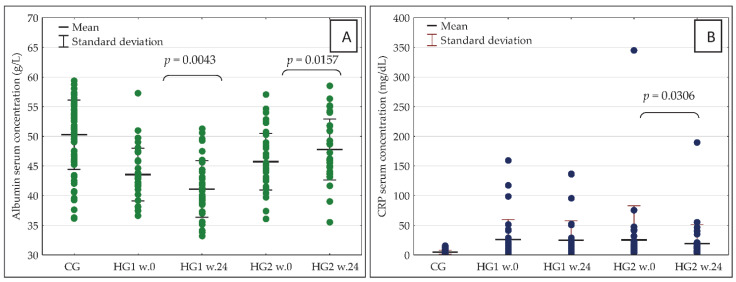
(**A**,**B**) Serum concentration of albumin (**A**) and CRP (**B**) in the studied groups of participants at baseline (week 0) and after 24 weeks of the dietary intervention. CRP—C-reactive protein; w.0—week 0; w.24—week 24; other abbreviations as in Figure 2.

The observed changes in nutrient intake were accompanied by significant changes in blood concentrations of the important markers of nutritional status and inflammation—albumin and CRP. In HG2, there was a significant increase in blood albumin concentration as well as a decrease in CRP concentration, while in HG1 there was a significant decrease in serum albumin concentration with no significant change in CRP concentration (Figure 4A,B).

In week 0, there were no significant differences between HG1 and HG2 in the serum concentration of either albumin or CRP, but some differences were recorded in albumin concentration after 24 weeks of the nutritional intervention. More specifically, the concentration was significantly higher in HG2 (*p* = 0.0000) than in HG1. In HG1, in comparison with CG, the serum concentrations of albumin were significantly lower both in week 0 (*p* = 0.0000) and in week 24 (*p* = 0.0000). Additionally, in HG2 vs. CG, the serum concentrations of albumin were significantly lower in week 0 (*p* = 0.0001) as well as in week 24 (*p* = 0.0340). In week 0 as well as in week 24, the serum concentrations of CRP were significantly higher both in HG1 and HG2 than in CG (*p* = 0.0000 in all cases).

**Figure 5:**
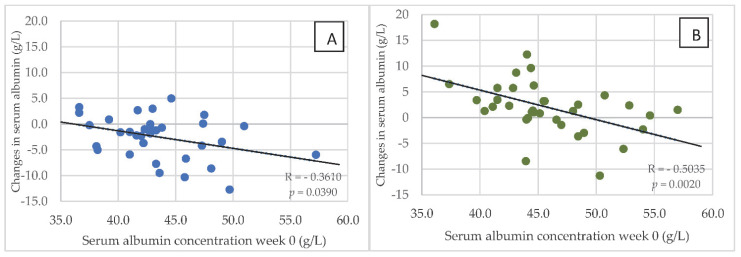
(**A**,**B**) Relationship between the serum concentration of albumin at baseline (in week 0) and changes in this parameter after 24 weeks of the nutritional intervention in HG1 (**A**) and HG2 (**B**). Abbreviations as in Figure 2.

In both groups of hemodialysis patients, serum concentrations of albumin in week 0 correlated negatively with changes in this parameter after 24 weeks of the dietary intervention. Figure 5A,B are reflective of these changes. In addition, in both groups of dialysis patients, the most marked changes associated with the reduction in serum albumin after 24 weeks were observed in those with the highest levels in week 0. However, in HG1, an increase in serum albumin was only observed in 7 patients, while this was seen in 25 patients in HG2. Serum concentration of albumin was below 4 g/L in: 6 patients—week 0 in HG1; 3 patients—week 0 in HG2; 16 patients—week 24 in HG1; 2 patients—week 0 in HG2. Serum concentration of CRP was above 10 mg/dL in: 26 patients—week 0 in HG1; 20 patients—week 0 in HG2; 23 patients—week 24 in HG1; 20 patients—week 0 in HG2.

## 4. Discussion

This was the first study to analyze long-term (6-month) effects of two kinds of nutritional intervention (with and without a meal served before dialysis) targeted at improving the nutritional status of hemodialysis patients. We also analyzed the dietary intake and nutritional status of a properly selected group of people from the general population who constituted the control group. Particular attention was paid to the development of dietary recommendations for each patient, especially bearing in mind energy, protein and fat demand, as well as the dietary content of potassium, sodium and phosphorus. For each patient, a dietary recommendation card was developed based on food exchangers with the album of the portion sizes presented in a 1:1 scale and sample menus. Additionally, patients received detailed information on the implementation of the dietary recommendations. Additionally, while collecting the 3-day dietary records of consumed foods and drinks (in weeks 5, 11, 17 and 23), patients were educated and received advice on how to improve their diet. Certified dieticians were responsible for education and preparation of individual nutritional programs. The above-mentioned aspects can be considered as the strengths of our research.

A very beneficial effect of the nutritional intervention applied was recorded in HG2, which was connected with an increase in the serum concentrations of albumin as well as a decrease in CRP, without significant changes in body mass, fat free mass and total body water. In contrast, in HG1, a significant increase in body mass occurred without changes in CRP, fat free mass and total body water but, unfortunately, a decrease in the serum concentration of albumin was observed. While an increase in the rates of survival accompanied by increases in the mean serum albumin and creatinine concentrations after starting IDPN or intradialytic oral nutritional supplementation were shown in numerous studies [27,28,29,30,31,32,33,34,39], the favorable impact of nutritional intervention in HG2 on the serum concentration of albumin and CRP was, to the best of the authors’ knowledge, studied for the first time. The authors were able to prove the theory formulated in 2001 by Kaysen et al., who suggested that it might be possible to modulate the effects of inflammation intentionally by increasing protein and calorie intake [10].

In both groups, it was also characteristic that gradual increases in energy and protein intake were observed but in HG1 this mainly occurred on Sundays and in HG2 on the days with dialysis. Bearing in mind that all patients were dialyzed three times a week, on average, patients in HG1 consumed 2622 kcal fewer, and those in HG2 3094 kcal fewer, per week than those in CG. Differences in energy intake between hemodialysis patients and CG in the study were at a similar level to those observed by Laville and Foque [26] where patients treated with hemodialysis consumed only 80% of the daily food rations and their energy deficiency was 2800 kcal per week. In weeks 17 and 23 of the nutritional intervention, differences in energy intake (analyzed in weekly terms), compared to CG, were at a similar level in both groups—in HG1 1778 kcal and in HG2 1835 kcal. Nevertheless, the beneficial effect in the form of an increase in serum albumin and a decrease in CRP was only observed in HG2. This may be related to the fact that in HG2, in weeks 17 and 23 compared to week 0, there was the greatest increase in energy intake (+271 kcal/day) and protein intake (+11.3 g/day). Serving a meal just before the start of dialysis may have reduced the intensification of catabolic processes associated with the dialysis procedure, as indicated by protein metabolism studies in which stable isotopes were used. 

It was shown that the introduction of IDPN or enteral nutrition during dialysis would maintain positive net metabolism (the anabolic advantage over catabolism) in both skeletal muscles and throughout the body. Veeneman et al. [27] examined the effects of consumption of six liquid portions (yoghurt, cream and milk protein powder) every 30 min on the whole-body protein metabolism (a method with primed constant infusion of L-[1-13C] valine) in hemodialysis patients on the days without dialysis and during dialysis. The total nutritional value of all portions was as follows: energy of about 1100 kcal, protein 46 g, carbohydrate 63 g and fat 75 g. It was shown that, on a non-dialysis day, protein balance was negative after an overnight fast. Consumption of the above-mentioned portions resulted in a positive whole-body protein balance. During dialysis, consumption of the same portions resulted in a positive protein balance to the same degree as on a non-dialysis day. In this study protocol, the authors used a protein intake of 0.6 g/kg, which accounted for about 50% of the daily protein demand. However, the authors assume that this amount of protein might be excessive for a hemodialysis patient during a 4 h dialysis session. In another study on protein metabolism during and after the hemodialysis procedure, IDPN and enteral nutrition with oral supplementation of industrial a ready to eat liquid diet, were applied. More specifically, IDPN was started 30 min after the hemodialysis initiation and continued until the end of the procedure. Enteral nutrition was administered as three equal meals: 0.5 h, 1.5 h and 2.5 h after the start of dialysis. The nutritional value of the IDPN protocol was as follows: energy 1258 kcal, protein 59 g, lipids 26 g, carbohydrates 197 g and the enteral nutrition protocol included: 1090 kcal, 57 g, 48 g and 109 g, respectively. During hemodialysis, the net whole-body and skeletal muscle protein metabolisms were highly positive compared to CG without meals. Additionally, in the post-hemodialysis phase, the positive net skeletal muscle metabolism persisted for enteral nutrition but not for IDPN [28]. These results clearly demonstrate that enteral nutrition can compensate adequately for the catabolic effects of the hemodialysis procedure and is an excellent strategy in the prevention and treatment of malnutrition. 

Our study revealed that a meal served just before the dialysis procedure, with a mean nutritional value as follows: energy 416 ± 23 kcal, protein 16.4 ± 0.6 g, fat 14.2 ± 1.6 g and total carbohydrates 58.2 ± 5.6, probably had a similar effect as observed by Pupim et al. [28], and was connected with a significant improvement in nutritional status in HG2. Given the fact that the demand for protein in patients treated with hemodialysis is at a level of 1.1 g/kg/d and the usual intake is lower than 1.0 g/kg/d [40], it is suggested that an average dialysis patient needs an additional supplementation of about 0.2 g/kg/d of protein [41]. Therefore, our proposed meals served before dialysis contained about 16 g of protein (0.22 g/kg/d). 

A significant increase in energy and protein intake on the days with dialysis was not achieved in HG1. Many different factors might have contributed to this, such as the long distance between the place of residence and the dialysis station, which is related to the fact that, on the days with dialysis, it takes a lot of time for patients to move to and from the dialysis center. Most patients are transported in groups of several people by ambulance, which further increases the time of travel. In addition, some patients feel unwell and are tired after the dialysis procedure, and it becomes impossible for them to prepare and consume attractive meals with an adequate nutritional value on that day. In this situation, the proposed solution of serving an attractive meal before the dialysis procedure is something that many patients appreciate, especially as it has had a measurable effect of improving their nutritional status and reducing the severity of inflammation. 

For the above reasons, the authors have used the acronym MEAM for the proposed nutritional intervention. It stands for Malnutrition—Eat Additional Meal and is related to the need to eat an additional meal before the dialysis procedure. The word MEAM is also defined as a contagious pattern of information with which the minds of others are “infected,” leading to individuals changing their behavior and promoting a particular behavior pattern [42]. Based on this definition, MEAM, referring to the promotion of eating an appropriate meal before dialysis, can be a valuable tool in combating malnutrition. 

Another aspect that has been discussed extensively in the literature relates to meal administration during dialysis and refers to the problem of intradialytic hypotension (IDH). The definition of IDH is not standardized. However, most definitions take into account either a relative or an absolute decline in blood pressure, as well as the presence of specific symptoms. Based on estimates, IDH occurs in 10–12% of all treatments, being higher in the group of elderly patients and in patients suffering from diabetes [43,44]. In addition, IDH can lead to premature termination of the hemodialysis session, which will result in the insufficient clearance of the patient’s blood [45]. However, there are some methods to prevent intradialytic hypotension, including blood volume monitoring, intradialytic temperature controls, ultrafiltration modulation, sodium profiling and modeling, alteration of the dialysate electrolyte composition, as well as pharmacological interventions [44,46,47]. 

In an interesting study, nephrologists in the USA were asked the following question: “why are there no meal trays for patients during hemodialysis treatment?” The most common causes were reported as follows: the risk of postprandial hypotension, the risk of choking or aspiration, infection control and hygiene issues, including fear of fecal–oral transmission of such diseases as hepatitis A, as well as staff burden and distraction [41]. During the International Society of Renal Nutrition and Metabolism Conference in Germany, clinicians were asked about their practices during hemodialysis. The results were surprising—about 85% of the surveyed clinics allowed patients to eat during dialysis, 65% actively encouraged patients to eat and 73% of the clinics provided food during the dialysis procedure. The results of this study indicate that eating during the dialysis procedure is common in many countries, and most of the proposed negative consequences of nutrition during treatment are rarely observed [48]. In most European and South East Asian countries, meals are routinely given to patients treated with hemodialysis. For example, in Germany patients eat during hemodialysis and have significantly higher serum albumin levels and greater survival rates than patients in the USA, where eating during dialysis is not allowed [49]. It is believed that a monitored serving of meals during dialysis is possible, and may constitute a cost-effective and patient-friendly strategy despite concerns such as postprandial hypotension, a risk of aspiration, infection control, hygiene and dialysis staff load [21].

In addition, bearing in mind the risk aspects associated, inter alia, with IDH, we decided to serve a meal immediately before dialysis and not during the procedure. We also ensured that the meal was easily digestible, not too rich (energy 416 kcal, protein 16.4 g) and not high in fat and fiber, which could increase the risk of gastrointestinal complaints and IDH. With respect to the obtained significant increase in the serum albumin concentration in HG2 and a decrease in CRP, the nutritional value of the served meal was sufficient to obtain this beneficial effect. With regard to the protein pool in the served meal, it was the amount that should compensate losses during the dialysis procedure. It was shown that 10–13 g of net amino acids are lost through the dialyzer during a standard hemodialysis procedure [50,51]. 

Our study has some limitations, which include: the lack of a sample size justification in terms of serum albumin and CRP concentrations (these calculations were made only for prognostic markers of malnutrition, which were included in the panel of our research), participants did not fully adhere to dietary recommendations (these reasons are discussed above), patients were not rigorously categorized according to their cause of end stage renal disease or used medications and patients sometimes did not eat the entire meal served before dialysis.

## 5. Conclusions

The nutritional intervention, referred to as the MEAM diet, which included dietary recommendation cards with individually calculated nutritional values, an album of portion sizes, education and easily digestible meals served before the dialysis procedure (energy 416 ± 23 kcal, protein 16.4 ± 0.6 g, fat 14.2 ± 1.6 g, and total carbohydrates 58.2 ± 5.6 g), was connected with an increase in the serum concentration of albumin and a decrease in CRP. The results of this study indicate the need not only for deeper education of hemodialysis patients in the area of their diet, but also for undertaking further research and discussions on the possibility of ensuring adequate meals for hemodialysis patients before the dialysis procedure.

## Figures and Tables

**Table 1:** General characteristics of the study participants (means ± SD or *n*, %).

Variables	CG	HG1	HG2
Sex: men/women (*n*)	38/32	21/14	18/17
Sex: men/women (%)	54.3/45.7	60.0/40.0	51.4/48.6
Age (years)	54.4 ± 7.0	59.7 ± 11.5	58.1 ± 15.7
Height (cm)	170.4 ± 8.9	168.1 ± 9.5	169.8 ± 10.5
BMI (kg/m^2^)	26.7 ± 4.4	26.6 ± 4.9	25.2 ± 4.9

CG—control group; HG1—patients who were to comply with the dietary recommendations without a meal served before dialysis; HG2—patients who were to comply with the dietary recommendations with a meal served before dialysis.

**Table 2:** Body mass and body composition of the study participants (means ± SD).

Variables	Stage/Week	CG	HG1		HG2	
			BD	AD	BD	AD
Body mass (kg)	I/0	77.9 ± 15.9	74.9 ± 19.4	74.5 ± 19.0	71.9 ± 13.2	71.3 ± 14.2
	II/12		75.2 ± 19.7	75.3 ± 18.9	71.7 ± 13.0	71.5 ± 14.3
	II/24		75.7 ± 19.5	75.4 ± 18.9	71.6 ± 13.0	71.2 ± 14.0
	*p*#		0.4325	0.0161	0.5541	0.6689
	*p*##		0.0205	0.0042	0.3232	0.5662
Fat free mass (kg)	I/0	57.2 ± 11.4	59.2 ± 13.1	55.9 ± 13.0	55.9 ± 9.1	52.4 ± 8.6
	II/12		59.7 ± 13.3	56.1 ± 13.0	56.3 ± 10.0	53.2 ± 9.6
	II/24		59.3 ± 13.4	55.9 ± 13.0	56.5 ± 9.7	53.0 ± 9.1
	*p*#		0.4709	0.5982	0.4193	0.0715
	*p*##		0.8279	0.8967	0.1714	0.3361
Total body water (kg)	I	40.4 ± 7.9	42.2 ± 9.6	39.2 ± 9.1	39.7 ± 6.8	36.9 ± 6.2
	II/12		42.7 ± 10.0	39.4 ± 9.2	40.1 ± 7.6	37.7 ± 7.3
	II/24		42.4 ± 10.0	39.2 ± 9.1	40.1 ± 7.2	37.2 ± 6.5
	*p*#		0.3170	0.5807	0.3665	0.0467
	*p*##		0.6796	0.9092	0.3030	0.5298

*p*#—baseline value (week 0) compared with the value after 12 weeks in the same group; *p*##—baseline value (week 0) compared with the value after 24 weeks in the same group; BD—measurements performed before the dialysis procedure; AD—measurements performed after the dialysis procedure; other abbreviations as in Table 1.

**Table 3:** Daily energy (kcal/kg/day) and protein (g/kg/day) intake in the studied groups of participants (means ± SD).

Stage/Weeks	Group	CG ^1^	WD	WOD	Su
Daily energy intake (kcal/kg/day)					
Stage I, week 0	CG	29.8 ± 7.7			
	HG1		23.4 ± 5.6 **	25.2 ± 6.1 *	25.3 ± 6.1 *
	HG2		23.4 ± 8.3 **	23.6 ± 10.1 **	23.0 ± 8.0 **
Stage II, mean values in weeks 5 and 11	HG1		23.6 ± 6.0 **	25.6 ± 7.9 *	27.4 ± 7.3
	HG2		25.4 ± 5.8 *	24.7 ± 6.6 *	25.4 ± 6.5 *
Stage II, mean values in weeks 17 and 23	HG1		24.7 ± 5.0 **	27.3 ± 6.0	28.8 ± 8.6
	HG2		27.1 ± 6.4	25.1 ± 6.3 *	24.7 ± 5.7 **
Daily protein intake (g/kg/day)					
Stage I, week 0	CG	1.36 ± 0.34			
	HG1		1.01 ± 0.26 **	1.06 ± 0.33 **	1.05 ± 0.30 **
	HG2		1.04 ± 0.45 **	1.07 ± 0.51 **	1.08 ± 0.35 **
Stage II, mean values in weeks 5 and 11	HG1		1.07 ± 0.26 **	1.13 ± 0.34 *	1.24 ± 0.34
	HG2		1.12 ± 0.30 **	1.11 ± 0.30 **	1.14 ± 0.31 *
Stage II, mean values in weeks 17 and 23	HG1		1.11 ± 0.32 *	1.22 ± 0.35	1.22 ± 0.35
	HG2		1.19 ± 0.30 *	1.14 ± 0.33 *	1.13 ± 0.39 *

^1^—data regarding CG are presented as mean values for 3 days; *—significant differences in comparison with values in CG, *p* ≤ 0.05; **—significant differences in comparison with values in CG, *p* ≤ 0.001. WD—a day with dialysis; WOD—a day without dialysis; Su—Sunday; other abbreviations as in Table 1.

## Data Availability

The data presented in this study are available on request from the corresponding author. The data are not publicly available due to privacy or ethical restrictions.

## Supplementary Materials

The following supporting information can be downloaded at: https://www.mdpi.com/article/10.3390/nu14245352/s1, Table S1. Daily energy and selected nutrient intake in the studied groups of participants.
