# Supplementary material for: Effects of the Malnutrition—Eat Additional Meal (MEAM) Diet on the Serum Levels of Albumin and C-Reactive Protein in Hemodialysis Patients
Source: Nutrients. 2022 Dec 16;14(24):5352. doi: 10.3390/nu14245352 (PMC9782191; doi:10.3390/nu14245352)
Supplement: Supplementary file 1 [file nutrients-14-05352-s001.zip › nutrients-2064869-supplementary.pdf]

Table S1. Daily energy and selected nutrients intake in the studied groups of participants (means ± SD)

Variables	Groups	CG[1]	Stage I, week 0			Stage II, mean values from weeks 5 and 11			Stage II mean values from weeks 17 and 23		
			WD	WOD	Su	WD	WOD	Su	WD	WOD	Su
Energy (kcal/day)	CG	2060 ± 493									
	HG1		1611 ± 422**	1742 ± 489*	1739 ± 455*	1626 ± 429**	1761 ± 559*	1880 ± 496	1696 ± 364**	1855 ± 310*	1989 ± 667
	HG2		1609 ± 580**	1636 ± 703**	1591 ± 547**	1752 ± 378*	1701 ± 429**	1747 ± 413*	1880 ± 418	1736 ± 413*	1737 ± 436*
Energy (kcal/kg/day)	CG	29.8 ± 7.7									
	HG1		23.4 ± 5.6**	25.2 ± 6.1*	25.3 ± 6.1*	23.6 ± 6.0**	25.6 ± 7.9*	27.4 ± 7.3	24.7 ± 5.0**	27.3 ± 6.0	28.8 ± 8.6
	HG2		23.4 ± 8.3**	23.6 ± 10.1**	23.0 ± 8.0**	25.4 ± 5.8*	24.7 ± 6.6*	25.4 ± 6.5*	27.1 ± 6.4	25.1 ± 6.3*	24.7 ± 5.7**
Protein (g/day)	CG	94.7 ± 25.2									
	HG1		69.4 ± 19.1**	72.9 ± 23.9**	72.4 ± 22.4**	73.6 ± 17.6**	77.7 ± 22.5*	84.6 ± 23.5	75.1± 18.8**	82.7 ± 17.9*	83.7 ± 22.7*
	HG2		71.8 ± 32.5**	74.1 ± 35.8*	75.0 ± 25.5**	77.7 ± 20.7**	76.9 ± 19.7**	78.2 ± 18.5*	82.9 ± 21.1*	79.1 ± 22.5*	79.2 ± 28.3*
Protein (g/kg/day)	CG	1.36 ± 0.34									
	HG1		1.01 ± 0.26**	1.06 ± 0.33**	1.05 ± 0.30**	1.07 ± 0.26**	1.13 ± 0.34*	1.24 ± 0.34	1.11 ± 0.32*	1.22 ± 0.35	1.22 ± 0.35
	HG2		1.04 ± 0.45**	1.07 ± 0.51**	1.08 ± 0.35**	1.12 ± 0.30**	1.11 ± 0.30**	1.14 ± 0.31*	1.19 ± 0.30*	1.14 ± 0.33*	1.13 ± 0.39*
Fat (g/day)	CG	61.1 ± 20.9									
	HG1		48.0 ± 18.9*	55.2 ± 21.7	55.4 ± 22.9	48.5 ± 17.7*	50.5 ± 23.7*	55.1 ± 22.1	45.3 ± 14.5**	51.3 ± 17.0*	50.3 ± 19.8*
	HG2		48.7 ± 22.7*	48.3 ± 28.4*	46.4 ± 19.2**	56.0 ± 15.1	49.6 ± 21.8*	50.9 ± 17.8*	59.3 ± 16.2	51.8 ± 22.5*	50.7 ± 18.8*
Sodium (mg/day)	CG	3958 ± 1291									
	HG1		3272 ± 1155*	3599 ± 1316**	3649 ± 1292	3545 ± 1241	3306 ± 879*	3820 ± 1198	3416 ± 1288*	3369 ± 782*	3649 ± 1274
	HG2		3460 ± 1381	3331 ± 1420*	3816 ± 2061	3526 ± 854	3417 ± 1037*	3743 ± 1373	3678 ± 1063	3315 ± 911*	3468 ± 1085
Potassium (mg/day)	CG	3847 ± 1147									
	HG1		2312 ± 859**	2543 ± 1038**	2444 ± 852**	2518 ± 977**	2561 ± 957**	2870 ± 1205**	2415 ± 976**	2820 ± 761**	2924 ± 1296**
	HG2		2372 ± 1251**	2534 ± 1299**	2679 ± 1021**	2333 ± 864**	2581 ± 898**	2722 ± 740**	2725 ± 1196**	2567 ± 711**	2684 ± 890**
Phosphorus (mg/day)	CG	1474 ± 441									
	HG1		918 ± 255**	943 ± 351**	949 ± 330**	1023 ± 256**	1098 ± 386**	1123 ± 353**	1038 ± 323**	1156 ± 263**	1237 ± 503*
	HG2		949 ± 456**	1037 ± 505**	959 ± 360**	1019 ± 317**	1004 ± 272**	1039 ± 282**	1081 ± 348**	1061 ± 369**	1012 ± 352**
Calcium (mg/day)	CG	744 ± 295									
	HG1		398 ± 208**	424 ± 315**	413 ± 232**	473 ± 260**	515 ± 380*	444 ± 169**	480 ± 217**	555 ± 245*	574 ± 310*

	HG2		435 ± 309**	459 ± 335**	352 ± 211**	368 ± 189**	384 ± 186**	370 ± 179**	397 ± 230**	440 ± 278**	381 ± 161**
Iron (mg/day)	CG	13.24 ± 3.56									
	HG1		8.85 ± 3.05**	9.74 ± 3.70**	9.54 ± 3.08**	9.27 ± 3.66**	9.61 ± 3.05**	10.71 ± 3.50**	9.76 ± 4.62**	9.76 ± 1.90**	11.63 ± 5.16
	HG2		8.55 ± 3.48**	8.58 ± 3.93**	9.34 ± 3.28**	9.29 ± 2.90**	9.79 ± 4.39**	9.58 ± 2.22**	10.02 ± 3.79**	10.06 ± 3.76**	9.08 ± 2.26**
Zinc (mg/day)	CG	11.35 ± 3.29									
	HG1		8.10 ± 3.20**	8.57 ± 2.57**	8.83 ± 3.07**	8.24 ± 2.59**	8.55 ± 2.28**	9.56 ± 3.38*	8.60 ± 2.23**	9.12 ± 1.67**	9.58 ± 2.98*
	HG2		8.13 ± 3.83**	7.81 ± 3.62**	8.62 ± 3.18**	8.29 ± 2.57**	8.31 ± 2.44**	8.78 ± 2.08**	8.69 ± 2.61**	8.74 ± 2.28**	8.42 ± 2.26**
Magnesium (mg/day)	CG	351.3 ± 104.3									
	HG1		220 ± 105**	229 ± 89**	234 ± 106**	227 ± 84**	221 ± 67**	255 ± 89**	225 ± 77**	244 ± 52**	259 ± 89**
	HG2		215 ± 98**	217 ± 75**	234 ± 92**	225 ± 77**	225 ± 65**	246 ± 81**	232 ± 70**	251 ± 78**	233 ± 66**
Vitamin A (µg retinol eq.)	CG	1233 ± 811									
	HG1		799 ± 641*	1004 ± 769	1048 ± 974	986 ± 1097	1043 ± 480	1154 ± 766	673 ± 289**	925 ± 455*	1060 ± 696
	HG2		735 ± 452*	855 ± 650*	1247 ± 1302	931 ± 1220	880 ± 611*	785 ± 406*	753 ± 378*	897 ±556*	946 ± 621
Vitamin E (mg α-tocopherol eq.)	CG	9.22 ± 5.43									
	HG1		4.98 ± 2.60**	5.93 ± 2.80*	5.51 ± 2.85**	5.17 ± 2.26**	5.98 ± 3.25*	5.60 ± 1.79**	5.48 ± 2.29**	6.12 ± 1.57*	5.56 ± 2.24**
	HG2		5.20 ± 2.52**	5.62 ± 3.52**	5.34 ± 2.92**	7.85 ± 2.55	6.24 ± 2.90*	5.70 ± 2.24**	6.96 ± 2.17	6.52 ± 4.20*	5.63 ± 3.12**
Vitamin B1 (mg/day)	CG	1.35 ± 0.53									
	HG1		0.94 ± 0.41**	1.04 ± 0.41*	1.01 ± 0.48*	1.03 ± 0.43*	0.95 ± 0.31**	1.19 ± 0.61	1.07 ± 0.33*	1.20 ± 0.35	1.14 ± 0.42
	HG2		1.02 ± 0.48*	0.96 ± 0.56**	1.11 ± 0.50*	0.96 ± 0.35**	1.13 ± 0.46*	1.18 ± 0.38	1.18 ± 0.45	1.25 ± 0.45	1.18 ± 0.62
Vitamin B2 (mg/day)	CG	1.88 ± 0.69									
	HG1		1.14± 0.34**	1.26 ± 0.49**	1.30 ± 0.48**	1.43 ± 0.79*	1.44 ± 0.53*	1.45 ± 0.52*	1.40 ± 0.71*	1.54 ± 0.36*	1.65 ± 0.81
	HG2		1.10 ± 0.46**	1.30 ± 0.67**	1.20 ± 0.43**	1.34 ± 0.53**	1.44 ± 1.22*	1.33 ± 0.38**	1.43 ± 0.90*	1.45 ± 0.90*	1.28 ± 0.51**
Vitamin B6 (mg/day)	CG	2.03 ± 0.82									
	HG1		1.36 ± 0.50**	1.38 ± 0.57**	1.36 ± 0.55**	1.44 ± 0.51**	1.37 ± 0.51**	1.70 ± 0.71*	1.70 ± 2.12	1.55 ± 0.42*	1.81 ± 1.17
	HG2		1.60 ± 1.30	1.43 ± 0.66**	1.59 ± 0.63*	1.43 ± 0.46**	1.56 ± 0.56*	1.65 ± 0.48*	1.57 ± 0.52*	1.45 ± 0.46**	1.57 ± 0.63*
Vitamin B12 (µg/d)	CG	5.84 ± 4.96									
	HG1		2.92 ± 2.82*	2.66 ± 1.60**	2.64 ± 1.54**	2.96 ± 1.65**	3.10 ± 1.46*	3.59 ± 2.71*	2.81 ± 1.81**	3.27 ± 1.78*	3.07 ± 1.53*
	HG2		2.58 ± 2.08**	2.82 ± 2.37**	2.28 ± 1.16**	3.02 ± 1.74*	2.29 ± 1.25**	2.46 ± 1.26**	2.22 ± 1.34**	2.23 ± 1.04**	2.24 ± 0.98**
Vitamin PP (mg/day)	CG	24.45 ± 10.37									
	HG1		14.6 ± 6.2**	13.9 ± 5.3**	14.4 ± 6.4**	15.5 ± 5,9**	14.7 ± 6.8**	18.5 ± 9.3*	17.1 ± 11.3*	16.8 ± 5.8**	20.5 ± 13.3
	HG2		16.8 ± 9.3**	15.9 ± 9.5**	17.8 ± 7.7*	16.4 ± 5.6**	17.9 ± 6.8**	18.0 ± 5.9*	19.6 ± 8.8*	17.1 ± 6.3**	17.9 ± 8.4*
Vitamin C (mg/day)	CG	115.7 ± 74.8									

	HG1		45.2 ± 34.3**	60.1 ± 61.5**	45.1 ± 32.9**	48.5 ± 33.0**	57,5 ± 45.0**	69.8 ± 76.0*	56.1 ± 40.7**	64.4 ± 37.9**	62.4 ± 44.0**
	HG2		60.3 ± 59.3**	63.3 ± 45.6**	68.8 ± 58.0*	75.7 ± 44.0*	71.6 ± 56.7*	75.7 ± 55.4*	57.6 ± 40.6*	68.8 ± 64.0*	59.7 ± 41.8**
Folate (μg/d)	CG	328.2 ± 149.4									
	HG1		187 ± 71**	217 ± 63**	201 ± 60**	233 ± 135*	226 ± 74**	241 ± 73*	236 ± 119*	248 ± 47*	253 ± 72*
	HG2		198 ± 82**	210 ± 92**	222 ± 94**	261 ± 96*	222 ± 74**	233 ± 58**	256 ± 165*	232 ± 120*	212 ± 65**
Cholesterol (mg/day)	CG	358 ± 140									
	HG1		258 ± 151*	264 ± 161*	333 ± 227	294 ± 213	319 ± 165	351 ± 242	235 ± 116**	302 ± 172	338 ± 200
	HG2		198 ± 82**	248 ± 175**	287 ± 158*	278 ± 154*	246 ± 118**	320 ± 156	290 ± 165*	272 ± 174*	273 ± 164*

[1] - data from CG are presented as mean value from 3 days, * - significant differences in comparison with value in CG, $p \leq 0.05$; ** - significant differences in comparison with value in CG, $p \leq 0.001$;

Abbreviation: CG – control group; HG1 - patients who were comply with the dietary recommendations without meal served before dialysis; HG2 - patients who were comply with the dietary recommendations with meal served before dialysis; WD – days with dialysis procedure; WOD - days without dialysis procedure; Su – Sunday
